# Supplementary material for: Shoulder girdle formation and positioning during embryonic and early fetal human development
Source: PLoS One. 2020 Sep 11;15(9):e0238225. doi: 10.1371/journal.pone.0238225 (PMC7485900; doi:10.1371/journal.pone.0238225)
Supplement: Attachment — Submitted filename: Response to Reviewers.docx [file pone.0238225.s006.docx]

PONE-D-20-11726
The shoulder girdle formation and position in the embryonic and early fetal periods

Reply to the editor

1. they also suggested to have the manuscript edited for its English language, preferably by a native speaker or professional editing service.

The manuscript was edited for its English language, by professional editing service, Editage Corp.

2. As the manuscript deals with human embryos, more sensitive language should be used to avoid potential controversies and negative impact to the journal and the authors.

1) For instance, the text in lines 110-118 is strong and could be an issue for the journal. The specimens used are scientific and seem to be ethically collected, but the statement that "written consent was not obtained from all parents" needs to be clarified. If verbal consent was obtained and recorded from all parents with the approval of the IRB, then this statement may be deleted.

Because verbal consent was obtained and recorded from all parents with the approval of the IRB, the phrase "written consent was not obtained from all parents" was removed.

2) Words such as "abortion" or "aborted fetuses" should be avoided.

Words "abortion" or "aborted fetuses" was rephrased.

3) You should provide the approval number (from your ethics committee) to validate/demonstrate proper supervision of your study.

The approval number from your ethics committee to demonstrate proper supervision of our study was provided (E986, R0316).

2. Please amend your current ethics statement to address the following points:
a) Please explain why was written consent was not obtained
b) Did the ethics committees/IRBs approve this consent procedure?

c) You state "Written consent was not obtained from all parents." Did they provide verbal consent as described?

d) Were the samples anonymized and/or de-identified?

e)) Once you have amended this/these statement(s) in the Methods section of the manuscript, please add the same text to the “Ethics Statement” field of the submission form (via “Edit Submission”).

We amend our current ethics statement as indicated, in the Methods section of the manuscript, and added the same text to the “Ethics Statement” field of the submission form (via “Edit Submission”).

3. We note you have included a table to which you do not refer in the text of your manuscript. Please ensure that you refer to Table 1 in your text; if accepted, production will need this reference to link the reader to the Table.

Table 1 (Table 3 in the revised manuscript) was referred in the text of our manuscript.

Reply to Reviewer #1

1. Line 22-3: unclear what is meant by this sentence

The sentence was amended as follows;

However, such data are limited except for information on the axial position of the scapula.

2. Line 32: by "height", do you mean axial position? Fix throughout manuscript.

Height was reworded using the term “axial position” through out the manuscripts.

3. Line 60: please define better "Th2-Th7 height"

The phrase "Th2-Th7 height" was rephrased as “the axial position between the Th2 and Th7 vertebrae”.

4. Lines 66-70: can this sentence be written more concisely and without direct reference in the text to the figures by Lewis?

This sentence was written more concisely as follows;

Lewis (1902) [5] precisely described the morphogenesis of the upper limb in the embryonic period, including the skeleton, muscles, and nerves. The scapula and clavicle were illustrated as skeletal components.

5. Line 70: What is meant by "higher" - more cranial? Fix throughout by using cranial or caudal to indicate direction of shift.

The word “higher” was removed and the sentence was changed as follows;

Regarding the scapula, the author paid particular attention to the cranial position of the scapula in the initial blastemal and chondrogenous phases [5].

6. Line 110: please reference a table and provide one.

Table 1 was provided.

7. Line 170: please correct by writing "ventral tip"

The word “tip” was rephrased as “ventral tip” as indicated.

8. Lines 184-198: Can these measurements be made into a descriptive table?

Table 2 was provided.

9. Line 222: What is meant by "lowered" - Please fix throughout the text.

The sentence was changed as follows;

The scapula had low signal intensity in MRI images at the proximal part of the acromion connected to the scapular body at CS23 (N=3).

10. Line 243 (and others). It is best to have the authors provide the number of biological replicates next to each observation they make (e.g., N=2). This is an important point, which should also be provided in each figure legend.
The number of biological replicates next to each observation was provided in texts and each figure legend.

11.Line 288-290: Can this be defined better?
The sentence was changed as follows;

Particularly, the spa and glc of the scapula were located at the cranial side of the x and y axes, while the scapula body was located at the caudal side of the x and y axes from the ventral and lateral views.

12. Line 304: Instead of "was around" use "was at the approximate positional height of the C6 and TH1 verebrae". Please correct instances where the language use is too casual. Be more scientifically accurate.

The sentence was changed as follows;

The approximate axial position of the spa and ifa was at the C6 and Th1 vertebrae, respectively, in the early embryonic period (N=6).

The word “around” was reworded through out the texts.

13. Line 336: Remove "around" and say "between" but there are others as well.
The sentence was changed as indicated.

The angle of the segment spa-ifa on the y-axis from the lateral view (∠Sc1l) was between 100 and 110 degrees in the early embryonic period (N=5), and decreased to (90 and 100 degrees) in the late embryonic period (N=18).

14. Line 398: Say "Lewis (year)" - no need for WH.

The phrase was changed as indicated.

Line 401: Say "each skeletal form"

The phrase was changed as indicated.

Line 407: What is meant by "main part of the scapula"?

The phrase was rephrased as “the scapula body”.

Line 433: The general scientific audience will not recognize what th1 and ifa are, and so it is best to use their full names and what they refer to here. Please correct all instances in the Discussion. It is okay for these descriptors to be used in the Results section provided they are defined at the first use. This will help the reader follow the text.
Most of abbreviations used in Discussion were spell out

Line 465: write "humeral head"
The phrase was changed as indicated.

Reply to Reviewer #2:

1. The entire manuscript should be edited by a native speaker since it contains several grammatical and syntactical errors.

The manuscripts were subjected to the English language editing service (Editage).

2. The authors use a large number of acronyms, particularly with regards to various anatomical angles. It would be helpful to include a Table listing all the acronyms.

Table 2 listing all the acronyms were provided.

3. A better title of the manuscript could be: “Shoulder girdle formation and positioning during embryonic and early fetal human development”.

The title was changed as indicated.
